# Epigenetic Changes and Transcriptional Reprogramming Upon Woody Plant Grafting for Crop Sustainability in a Changing Environment

**DOI:** 10.3389/fpls.2020.613004

**Published:** 2021-01-12

**Authors:** Aliki Kapazoglou, Eleni Tani, Evangelia V. Avramidou, Eleni M. Abraham, Maria Gerakari, Stamatia Megariti, Georgios Doupis, Andreas G. Doulis

**Affiliations:** ^1^Department of Vitis, Institute of Olive Tree, Subtropical Crops and Viticulture (IOSV), Hellenic Agricultural Organization-Demeter (HAO-Demeter), Athens, Greece; ^2^Laboratory of Plant Breeding and Biometry, Department of Crop Science, Agricultural University of Athens, Athens, Greece; ^3^Laboratory of Forest Genetics and Biotechnology, Institute of Mediterranean Forest Ecosystems, Athens, Hellenic Agricultural Organization-Demeter (HAO-Demeter), Athens, Greece; ^4^Laboratory of Range Science, Faculty of Forestry and Natural Environment, Aristotle University of Thessaloniki, Thessaloniki, Greece; ^5^Department of Viticulture, Vegetable Crops, Floriculture and Plant Protection, Institute of Olive Tree, Sub-Tropical Crops and Viticulture, Hellenic Agricultural Organization-Demeter (HAO-Demeter) (fr. NAGREF), Heraklion, Greece

**Keywords:** grafting, rootstock, scion, woody species, epigenetics, transcriptional reprogramming, climate change, transgenerational inheritance

## Abstract

Plant grafting is an ancient agricultural practice widely employed in crops such as woody fruit trees, grapes, and vegetables, in order to improve plant performance. Successful grafting requires the interaction of compatible scion and rootstock genotypes. This involves an intricate network of molecular mechanisms operating at the graft junction and associated with the development and the physiology of the scion, ultimately leading to improved agricultural characteristics such as fruit quality and increased tolerance/resistance to abiotic and biotic factors. Bidirectional transfer of molecular signals such as hormones, nutrients, proteins, and nucleic acids from the rootstock to the scion and vice versa have been well documented. In recent years, studies on rootstock-scion interactions have proposed the existence of an epigenetic component in grafting reactions. Epigenetic changes such as DNA methylation, histone modification, and the action of small RNA molecules are known to modulate chromatin architecture, leading to gene expression changes and impacting cellular function. Mobile small RNAs (siRNAs) migrating across the graft union from the rootstock to the scion and vice versa mediate modifications in the DNA methylation pattern of the recipient partner, leading to altered chromatin structure and transcriptional reprogramming. Moreover, graft-induced DNA methylation changes and gene expression shifts in the scion have been associated with variations in graft performance. If these changes are heritable they can lead to stably altered phenotypes and affect important agricultural traits, making grafting an alternative to breeding for the production of superior plants with improved traits. However, most reviews on the molecular mechanisms underlying this process comprise studies related to vegetable grafting. In this review we will provide a comprehensive presentation of the current knowledge on the epigenetic changes and transcriptional reprogramming associated with the rootstock–scion interaction focusing on woody plant species, including the recent findings arising from the employment of advanced—omics technologies as well as transgrafting methodologies and their potential exploitation for generating superior quality grafts in woody species. Furthermore, will discuss graft—induced heritable epigenetic changes leading to novel plant phenotypes and their implication to woody crop improvement for yield, quality, and stress resilience, within the context of climate change.

## Introduction

Grafting is an ancient agricultural propagation technique widely used to improve plant performance, in terms of yield, quality and resilience to abiotic and biotic stresses. It involves the merging of two genetically different plant parts, the rootstock, and the scion, in such a manner that the two parts join and grow as a single plant. Grafting is essentially dependent on the fundamental ability of wound healing in plants (Goldschmidt, 2014). Grafting is commonly used to improve production in woody fruit, nut crops (Warschefsky et al., 2016) as well as in non-woody vegetable crops (Abd Al-Razaq, 2019) and in order to influence scion performance in forest tree species (Jayawickrama et al., 1997) and in ornamental plants (Ambros et al., 2016). Important grafting applications include using rootstocks (a) to propagate plants that cannot be grown from seeds or cuttings (plants that do not set seed or have poor rooting ability), like certain cultivars of *Olea europaea* (Ayoub and Qrunfleh, 2006), (b) to control the juvenile period of the seedling (e.g., the prolonged delay in starting to bearing fruit observed in many *Citrus* genotypes (Warschefsky et al., 2016), (c) to replace the existing non-productive cultivar, or an old individual, with an improved one, capable of reliable and steady yield (Mudge et al., 2009). (d) To control canopy size and scion vigor, a practice widely used in apple cultivation (Fazio et al., 2015), (e) to repair rapidly a tree that has experienced significant damage either from natural causes or from management practices, and (f) to improve abiotic and biotic stress resistance characteristics (e.g., tristeza virus in citrus, *Phytophthora* on apple, *Meloidogyne* on peach, drought or phyloxera tolerance in grape).

Despite its widespread use, the molecular mechanisms underlying grafting are not fully understood although progress has been achieved in recent years in model and vegetable plants and recently in woody species as well. Transcriptional reprogramming and epigenetic changes seem to play crucial roles in the molecular mechanisms regulating rootstock-scion interactions and the development of the grafted plant. Epigenetics refers to stable and heritable changes in chromatin architecture that do not involve changes in the underlying DNA sequence but profoundly affect gene expression and impact cellular function. Epigenetic alterations are attained by three epigenetic mechanisms, namely, DNA methylation, post-translational histone modification and the action of non-coding RNA molecules which are either small RNAs (small interfering RNAs-siRNAs and micro RNAs-miRNAs) or long non-coding RNAs (long ncRNAs). Epigenetic regulation plays a major role in all aspects of plant development such as proper vegetative growth, successful reproduction, fruit development, yield, fruit quality, and tolerance to environmental stresses (Fujimoto et al., 2012; Kapazoglou et al., 2018). Moreover, understanding the vis ‘a-vis comparison of climate change impacts in relation to the impact of mitigation measures for agricultural and forestry and other land use is of significant importance in order to predict future food and energy sustainability (Van Meijl et al., 2018). For example, climatic changes are expected to exacerbate the negative impact of biotic stresses such as pathogens and pests. Losses in major crops and the role of crop protection has to be considered as a global strategy in order to safeguard future food needs (Oerke and Dehne, 2004). For instance, rising temperatures and rainfall changes are expected to increase water demand during the vegetative period, and in addition photodamage induced by solar radiation stress and high UV-B doses would be more detrimental for the Mediterranean crops (Doupis et al., 2011, 2013; Fares et al., 2017). Consequently, the effective abiotic stress assessment should focus on the development and evaluation of rootstocks that can influence scion growth and productivity under drought and light stress; particularly those rootstocks that can increase water conservation and those that can avoid photo bleaching by changes in leaf orientation and canopy structure. Considering the aforementioned biotic and abiotic constrains, emergence for better adaptation and selection as well as for reproduction of superior genotypes (e.g., through grafting or *in vivo* techniques) will have a significant impact in the future. Indeed, various studies nowadays focus on adaptation through grafting approaches for example cucumber salinity tolerance of cucumber (Elsheery et al., 2020), while a comprehensive recent review points out the significance of grafting for disease control, productivity and fruit quality (Belmonte-Urena et al., 2020). Creation through grafting of new superior genotypes will have a significant impact in order to achieve sustainable agricultural production and food security under the climatic changes which are evident nowadays. Understanding the molecular mechanisms underlying grafting with focus on the rootstock-scion interactions in woody species and the knowledge accumulated, thus far, with respect to the transcriptional and epigenetic effects associated with this process and the implications for woody crop improvement is an important task toward this direction.

## Molecular Mechanisms Underlying Grafting and the Epigenetic Component

**FIGURE 1:**
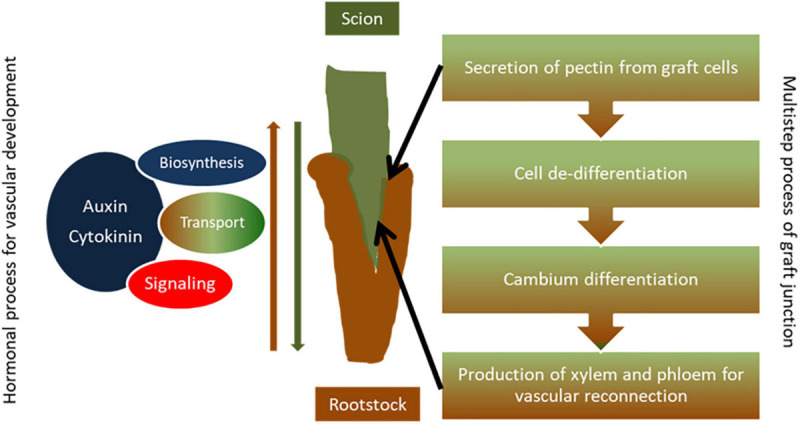
Simple diagram presentation of graft union formation. Establishment of a proper graft junction is a complex process which involves the secretion of pectin from graft cells to serve as an adherent for the two parts, cell de-differentiation and formation of callus at the grafting site to form a bridge between graft partners, and cambium differentiation and production of secondary xylem and phloem that enables vascular reconnection between the scion and the rootstock. Hormones such as auxin and cytokinin regulate the grafting process. Auxin accumulation takes place at the graft site above the graft junction and genes related to auxin biosynthesis, auxin transport, and signaling are induced during the grafting process in order to promote vascular development at the graft union. Similarly, gene networks involved in cytokinin biosynthesis and signaling as well as hormonal cross talk are activated toward efficient vascular formation, tissue reconnection, and graft development.

**FIGURE 2:**
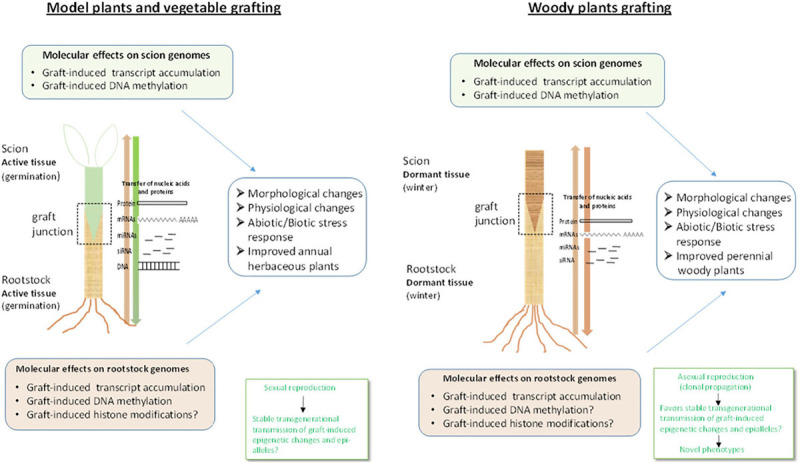
Molecular effects of grafting in vegetables and woody species. Grafting in vegetable plants involves active and rapidly grown tissues provided soon after germination whereas in woody species grafting involves dormant tissues. This implies that signaling and rootstock-scion interaction may also exhibit differences between the two (Gautier et al., 2019) at least in the initial stages of graft formation and development Transfer of coding (mRNA) and non-coding (miRNAs, siRNAs) RNAs along the graft, induction of transcript accumulation at gene-specific and genome-wide level and DNA methylation changes in the scions have been observed for both grafted vegetables and woody species. However, it not yet known if the same mobile molecules, for instance the same small RNA molecules and accompanying proteins, are transferred through the phloem in vegetable grafts and woody species grafts and under which conditions. In addition, graft-induced DNA methylation changes have not been shown, thus far, in woody species rootstocks whereas graft-induced epigenetic changes such as histone modifications have not been reported for either vegetable or woody species, as yet, although histone modification related genes have been found to display altered expression profiles. In addition, it is not known how these epigenetic modifications may vary between vegetables and woody plants species and the cross talk with hormonal networks in each case needs to be elucidated. Furthermore, it should be noted that unlike vegetables that are propagated by sexual reproduction, woody plants are clonally propagated by asexual reproduction. This would favor stable trans-generational transmission of graft-induced epigenetic changes such as DNA methylation, in woody crops, which may lead to phenotypic variation and ultimately to novel varieties with improved traits. The aspects described above suggest that although a plethora of information has been obtained regarding the molecular basis of grafting, the exact mechanisms underlying signaling and communication between rootstock and scion and graft-induced effects in either vegetables or woody plants remain unclear and present exciting challenges for future research.

Most of our knowledge on the molecular mechanisms of grafting has arisen from studies with model and vegetable species but in recent years studies on woody plants have been reported as well. Successful grafting requires a compatible and highly intricate interaction between the two parts of the graft, the scion, and the rootstock, which involves the concerted action of nutrient, hormonal, metabolic, transcriptional, and epigenetic pathways (Figures 1, 2). Establishment of a proper graft junction is a multistep process and hormones such as auxin, cytokinin, ethylene, gibberellin, and jasmonic acid play crucial roles in the scion-rootstock interaction (Melnyk and Meyerowitz, 2015; Melnyk et al., 2018; Nanda and Melnyk, 2018; Gautier et al., 2019; Sharma and Zheng, 2019). Homografts (same genotype used as scion and rootstock) present the highest grafting capacity but heterografts (different genotypes from the same species or different species) can also be successful depending on the phylogenetic distance between species and the particular genotype combinations. Accumulating evidence indicates short- or long-distance trafficking of signal molecules such as hormones, nutrients, proteins, and nucleic acids across the graft junction at cells adjacent to the graft interphase, or long range, at distant recipient tissue, with important implications to the success of the grafting and the development and performance of the scion (Albacete et al., 2015; Wang et al., 2017; Nanda and Melnyk, 2018; Gaut et al., 2019; Sharma and Zheng, 2019; Thomas and Frank, 2019).

Short-distance movement of DNA molecules was shown in early studies by Stegemann and Bock (2009). By employing transgenic and transplastomic tobacco grafting systems these authors were able to show movement of both nuclear and plastid DNA in cells adjacent to the graft interface at either side of the graft (Stegemann and Bock, 2009). Utilizing a similar system, movement of entire plastid genomes was demonstrated in reciprocal grafts of *Nicotiana tabacum*, *N. benthamiana*, and *N. glauca* (tree tobacco) and most notably, the transferred plastid DNA was stably inherited in regenerated plants pointing to evolutionary and agronomical implications (Stegemann et al., 2012). Nuclear genome transfer between rootstock and scion was also demonstrated in tobacco grafts (Fuentes et al., 2014).

Bi-directional transport of RNA molecules over long distances via the phloem of grafted as well as non-grafted plants has been well established over the last two decades (Lucas et al., 2001; Lough and Lucas, 2006; Mermigka et al., 2006; Kalantidis et al., 2008; Melnyk et al., 2011b; Haroldsen et al., 2012; Melnyk et al., 2018; Morris, 2018; Tamiru et al., 2018; Thomas and Frank, 2019; Wang et al., 2020). Protein-coding RNA movement across grafting partners was first reported in a *Cucurbitaceae* heterograft for *CmNACP*, a *Cucurbita maxima* (pumpkin) transcript (Ruiz-Medrano et al., 1999). *CmNACP* encodes a NAC-domain transcription factor involved in meristem formation and crucial for controlling organ boundaries and organ development. *CmNACP* mRNA was detected in the cucumber part of a cucumber (scion)-pumpkin (rootstock) graft, indicating graft–induced transfer from the pumpkin rootstock via the phloem to the cucumber scion (Ruiz-Medrano et al., 1999). In a similar manner, Xoconostle-Cázares et al. (1999) demonstrated that a mRNA molecule encoding the phloem protein CmPP16 moved along with its protein across the graft union and into the phloem of the cucumber scion which was detected also in stems, leaves, and floral scion tissues (Xoconostle-Cázares et al., 1999).

The physiological relevance of mRNA transport was rather obscure at first and started being delineated in later studies showing that movement of certain mRNA molecules from source to sink or the other way around is linked to important biological functions. For example, translocation of a homeobox fusion transcript from rootstock to scion caused changes in scion leaf morphology in tomato grafts (Kim et al., 2001). Transport of *BEL1* transcription factor transcripts (encoding regulators of tuber formation), from scion to roots enhanced tuber production in potato grafts (Banerjee et al., 2006) whereas a similar movement of *BEL11* and *BEL29* transcripts suppressed tuber growth in potato roots (Ghate et al., 2017). Similarly, movement of *AUX/IAA* transcripts (encoding regulators of auxin transport) to the root tip, modified tobacco root architecture in Arabidopsis/tobacco hetero-grafts (Notaguchi et al., 2012).

Massive long-distance transport of mRNA molecules in a series of homograft or heterograft systems also has been described. Utilizing Arabidopsis ecotype-specific SNPs, thousands of mRNAs were found to be transported between Arabidopsis graft partners and were differentially detected in response to nutritional conditions (Thieme et al., 2015). Over 3,000 transcripts moved from cucumber scion leaves to watermelon sink tissues in a tissue-specific manner as an early response to phosphate deficiency stress (Zhang et al., 2016), 138 transcripts moved from Arabidopsis rootstock to *Nicotiana benthamiana* scion (Notaguchi et al., 2015) and 183 transcripts migrated from *N. benthamiana* scions to tomato roots (Xia et al., 2018). It is thought that these migrating mRNAs attain a tRNA-like structure that confers stability, mobility, and translational capacity (Morris, 2018). Exciting new work has shown that cytosine methylation of mRNAs is an essential element for transport of mobile transcripts from shoot to root in Arabidopsis grafts and the translocation of a methylated TCPT transcript to root tissue had an effect on root growth (Yang et al., 2019). The physiological relevance of mobile transcriptomes along the graft partners has just started to be addressed (discussed in following sections) and it is expected to be further elucidated in future research (Morris, 2018).

Apart from protein-coded mRNA movement a plethora of studies during the last two decades has focused on the transport of non-coding small RNAs (short interfering RNAs-siRNAs and micro RNA-miRNAs) across graft segments. Small RNAs comprise a category of 21–24 nucleotides (nt) RNAs of different origins that are directed either to chromatin DNA loci in the nucleus, or target homologous mRNA molecules in the cytoplasm. Extensive studies have demonstrated the essential and instrumental role of small RNAs in all aspects of plant growth and development and in both abiotic and biotic stress responses (reviewed in Gohlke and Mosher, 2015; Lewsey et al., 2016; Li and Zhang, 2016; Banerjee et al., 2017; Kumar et al., 2017; Kumar and Sathishkumar, 2017; Li et al., 2017; Poltronieri et al., 2020; Wang et al., 2020).

Migration of 21-nt and mostly 24-nt small RNAs in homografted and heterografted plants had been demonstrated originally mostly in Arabidopsis, tobacco, *Solanaceae*, and *Cucurbitaceae* species (Molnar et al., 2010; Melnyk et al., 2011a; Haroldsen et al., 2012). Most importantly mobile small RNA molecules were found to exert a transcriptional or post-transcriptional gene silencing effect in graft partners either through RNA-directed DNA methylation (RdDNA) on targeted genomic loci (mainly for siRNAs) or via degradation of the corresponding mRNA target molecule (mainly for miRNAs) (Molnar et al., 2010; Melnyk et al., 2011a; Tamiru et al., 2018). Molnar et al. (2010) reported movement of both transgene-derived and endogenous small RNAs across the graft union in a shoot-to-root direction in Arabidopsis grafts and showed that the 24-nt siRNAs were able to direct DNA methylation at three sites in the genome of the recipient cells (Molnar et al., 2010). Furthermore, in a similar study, Lewsey et al. (2016) showed that the mobile siRNA signal migrating to the roots of an Arabidopsis graft, guided genome-wide DNA methylation events at thousands of loci of the recipient root genome predominately targeting transposable elements (TE) (Lewsey et al., 2016). These silencing effects have the potential to trigger gene expression changes impacting cellular functions ultimately leading to changes in the morphology and physiology of the grafted plant. Several reports have described long distance movement of miRNAs and their potential effects. In Arabidopsis miR399, miR827, miR2111 travel long-distance from shoot to root as a response to phosphate starvation (Huen et al., 2017). In *Lotus japonicus*, miR2111 translocates in a shoot-to-root direction to control rhizobial infection through post-transcriptional regulation of a key suppressor of symbiosis promoting increased nodulation (Tsikou et al., 2018).

In addition, graft-induced protein-coding RNAs and non-coding small RNAs have been detected in the scion or rootstock of vegetables (Ren et al., 2018; Miao et al., 2019; Wang et al., 2019a,b; Zhang et al., 2019a,b; Aslam et al., 2020; Garcia-Lozano et al., 2020; Spanò et al., 2020) as well as woody species grafts (Cookson et al., 2013; Corso et al., 2015; Kaja et al., 2015; Li et al., 2016; Chitarra et al., 2017; Pagliarani et al., 2017). Such graft-induced transcripts have a putative role in graft development, yield, fruit quality of scions and in response to abiotic or biotic factors (Ren et al., 2018; Zhang et al., 2019a,b; Garcia-Lozano et al., 2020; Spanò et al., 2020) (described in following sections).

In several other studies epigenetic effects have been reported in scions of homografts or hetero-grafts, although RNA movement has not been investigated in these systems, as yet. Utilizing methylation sensitive amplified polymorphism (MSAP) analysis in a *Cucurbitaceae* inter-species graft system, a significant DNA methylation increase was observed both in melon and cucumber scions heterografted onto pumpkin, as compared to the seed plants (Avramidou et al., 2014). Similarly, graft-induced alterations in global DNA methylation were evidenced in tomato, eggplant and pepper scions in a *Solanaceae* inter-species grafting system (Wu et al., 2013). Importantly, the DNA methylation changes could be inherited in the self-pollinating progeny, pointing to stable transfer of graft-induced epigenetic changes to the next generation (Wu et al., 2013). Furthermore, intra-species grafting in *Cucurbitaceae* induced significant DNA methylation changes in pumpkin scions which were associated with altered fruit morphology and metabolic profiles (Xanthopoulou et al., 2019). Recently such graft-induced epigenetic alterations were reported also in woody species. DNA methylation profiles in *Hevea brasiliensis* (rubber tree) heterografts were significantly altered depending on rootstock and rootstock-scion genotype combinations (Uthup et al., 2018) and DNA methylation polymorphisms were found between juvenile seedlings, grafts, and adult trees, in apple (Perrin et al., 2020). All the aforementioned studies indicate that grafting can trigger changes in epigenetic factors such as DNA methylation and small RNAs, potentially impacting important traits of agronomical relevance.

## Graft-Induced Transcriptional Reprogramming and Epigenetic Changes in Woody Species

Progress has been achieved in recent years with respect to the effects of grafting on the improvement of woody plants and the mechanisms underlying this process at the physiological, genetic, and epigenetic level. Employment of advanced—omics technologies has contributed greatly on the genome-wide aspects of rootstock-scion interactions, genotype compatibility, and graft-induced transcriptional and epigenetic effects. Some examples describing these investigations in woody species of high economic value are presented below and are summarized in Tables 1, 2.

**TABLE 1:** Examples of graft-induced transcriptional and epigenetic changes and physiological effects in woody species.

**Woody species**	**Graft-induced transcript/epi-genetic mark or mobile signal**	**Graft system**	**Tissue**	**Molecular response**	**Potential physiological change/agronomical trait**	**References**
Avocado (*Persea americana*)	miRNA	Seedling rootstocks and scions, Clonal rootstocks and mature scionwood (budwood)	Leaves (Scion)	miRNA induction in scion leaves	Transition from juvenile to flowering phase	Ahsan et al., 2019
Cassava (*Manihot esculenta*)	siRNAs	Non-transgenic scion/transgenic rootstock	Leaves (scion)	Translocation of siRNA from rootstock to scion, graft-induced systemic silencing	Resistance to CBSD virus	Yadav et al., 2011
Blueberry *(Vaccinium corymbosum)*	Hormonal Signaling	Non-transgenic scion/transgenic rootstock	Leaves (scion)	Phytohormones induced by transgenic blueberry (T) carrying a 35S-driven blueberry *FT* (*VcFT*-OX)	Flower induction	Song et al., 2019b
Grapevine *(Vitis vinifera)*	miRNA	Reciprocal heterografts Cabernet Sauvignon/M4	Leaves (scion) and roots (rootstock)	A series of conserved and novel miRNAs either in scion or rootstock are up- or down-regulated depending on drought conditions and rootstock	Differential drought-response	Pagliarani et al., 2017
Grapevine *(Vitis vinifera)*	mRNAs	Cabernet Sauvignon/41B Cabernet Sauvignon/1103 Paulsen	Leaves (scion)	Differential expression of genes associated with photosynthesis, secondary metabolism, hormonal signaling, stress response depending on rootstock	Vigor, productivity Biotic stress resistance	Chitarra et al., 2017
Grapevine *(Vitis vinifera)*	mRNAs	Reciprocal combinations of Pinot Noir and 1103 Paulsen	Roots (rootstock)	Differential graft-induced gene expression in roots depending on scion-rootstock combination	Root response	Gautier et al., 2020
Cherry (*Prunus avium*)	siRNA	Non-transgenic scion/transgenic rootstock	Leaves (scion)	Translocation of siRNA from rootstock to scion, graft-induced systemic silencing	Resistance to PNRSV virus	Song et al., 2013; Zhao and Song, 2014
Apple (*Malus domestica*)	miRNA	Heterografts with susceptible and resistant rootstocks	Leaves (scion)	miRNA induction in scion leaves	Resistance to fire blight	Kaja et al., 2015
Apple (*Malus domestica*)	miRNA	Three apple materials were analyzed: M9, Fuji, and Fuji/M9	Leaves (scion)	A regulatory network between miRNAs and mRNAs that is generated by grafting	Flower formation	An et al., 2018
Apple *(Malus domestica*)	DNA methylation	GDDH13 line grafted on scion cultivar “MM106”	Leaves (scion)	Differential DNA methylation	Mother plant DNA methylation pattern transmitted to graft	Perrin et al., 2020
Orange *(Citrus sinestis)*	DNA methylation	Valencia orange scion grafted on Rangpur Lime (RL) and Sunki Maravilha (SM), two rootstocks with different drought responses	Leaves (scion)	Differential DNA methylation under recurrent water stress	Increased “memory of stress” in the VO/SM combination	Neves et al., 2017
Tahiti acid lime *(Citrus latifolia* Tanaka)	DNA methylation	Tahiti acid lime grafted on RL and SM	Leaves (scion)	Differential DNA methylation under recurrent water stress	Similar physiological responses	Santos et al., 2020

**TABLE 2:** Mobile signals and their potential function in grafted Rosaceae plants.

**Graft-induced transcript or mobile signal**	**Target traits**	**Species**	**References**
MpSLR/IAA14	Unknown	*Malus domestica* (apple)	Kanehira et al., 2010
mdm-iR169a, mdm-iR160e, mdm-iR167b-g, mdm-iR168a,b	Fire blight resistance	*Malus domestica*	Kaja et al., 2015
miR156, miR172, miR159, miR171	Flower formation	*Malus domestica*	An et al., 2018
Unknown	Wooly apple aphid	*Malus domestica*	Sandanayaka et al., 2003
PbWoxT1/PbPTB3	Flower development and growth	*Pyrus betulaefolia* (pear)	Duan et al., 2016
MdGAI/PbGAI	Growth rate	*Pyrus betulaefolia, Malus domestica*	Xu et al., 2010; Zhang et al., 2012

### Grapevine

Viticulture is a vital sector of agriculture in many countries around the world. Global production of grape and wine amounts to 73.3 million tons of grape berry and 279 million hl of wine, annually (OIV statistical report on world vitiviniculture-http://www.oiv.int).

Grapevine grafting has been used widely to improve yield and quality and cope with the threat of diseases. The use of rootstocks is a common practice in most viticultural areas since the second half of nineteenth century in order to control infestation by phylloxera (*Daktulosphaira vitifoliae*), a soil-borne aphid that destroyed around four million of own-rooted vineyard hectares. The common commercial rootstocks currently used around the world were developed from native American *Vitis* species that have co-evolved with phylloxera and, as a result, they display a higher level of resistance (Mudge et al., 2009; Corso and Bonghi, 2014). Grapevine yield and quality of berry and wine depends on a variety of parameters such as genotype, rootstock, abiotic, and biotic factors, soil, and agricultural practices. A major factor affecting grapevine characteristics in relation to the environment is grafting onto appropriate rootstocks (Koundouras et al., 2008; Corso and Bonghi, 2014; Corso et al., 2015, 2016; Warschefsky et al., 2016; Peccoux et al., 2018; Wang et al., 2019b). A number of investigations have focused on the potential effects of scion/rootstock RNA movement along grapevine grafts as well as graft-induced transcriptional reprogramming with respect to rootstock specificity and plant performance. Using diagnostic SNPs from high throughput genome analyses to distinguish between scion and rootstock sequences more than 3,000 transcripts were found transported across graft junctions in field-or greenhouse-grown grafted grapevines (Yang et al., 2015). A large number of these sequences was associated with diverse biological functions and was highly enriched for genes involved in signal transduction cascades, metabolic activities, and stress responses implying they most likely influence whole plant development and physiology.

Graft-induced transcriptional reprogramming of mRNAs and miRNAs linked to particular scion/rootstock combinations have been described in several reports although their mobility aspect has not been characterized thus far. Graft-induced differential gene expression was reported in the shoot apex tissue of cv. Cabernet Sauvignon scions grafted onto either Riparia Gloire de Montpellier (RG) or Paulsen 1,103 rootstocks (Cookson and Ollat, 2013). Interestingly, these rootstocks conferred vigor effects and biomass accumulation to the scion which may be associated with the profound alterations in gene expression observed in scion shoots. The rootstocks induced expression of a multitude of genes which were enriched for chromatin modification genes (DNA methyltransferases and histone modifying genes), cell organization, hormonal signaling, and defense responses (Cookson and Ollat, 2013). Likewise, differential transcriptional responses related to secondary and jasmonate metabolism were reported for field-grown cv. Pinot Noir grafted onto a drought-sensitive rootstock (125AA) and a drought-tolerant rootstock (110R) upon drought stress. Berries of drought exposed Pinot Noir grafted on the drought-susceptible rootstock displayed increased induction of genes that are associated with the biosynthesis of primary and secondary metabolites affecting berry composition and wine quality (Berdeja et al., 2015). In addition, analysis of the effect of rootstock on Cabernet Sauvignon berries demonstrated that modulation of auxin-related genes and the rate of ripening varies according to the rootstock used in the graft (Corso et al., 2016).

Pagliarani et al. (2017) reported differential and drought stress-specific accumulation of miRNAs in auto-grafts and reciprocal heterografts of Cabernet Sauvignon and M4, a drought tolerant hybrid. Differential expression of novel and conserved miRNAs evidenced in all grafts depended on genotype combinations, tissue type, and drought stress conditions and graft directionality (Pagliarani et al., 2017).

An integrative physiological, metabolomics, and transcriptomic analysis using hetero-grafts of the southern Italian grapevine variety “Gaglioppo” highlighted the multiple effects of different rootstocks on the physiology, gene expression and metabolic pathways of the grafted plants (Chitarra et al., 2017). Significant differences in gene expression were evidenced in the leaves of “Gaglioppo” scions depending on rootstock genotype. Differentially Expressed Genes (DEGs) associated with photosynthetic processes, secondary metabolism, hormonal, stress, and signal transduction processes were upregulated in the scions of a Gaglioppo scions grafted on 41B rootstocks. Moreover, although less productive and less vigorous, Gaglioppo/41B exhibited increased resistance to pathogen infection by *Plasmopara viticola* as compared to the susceptible combination Gaglioppo/1103P. Interestingly, the 41B rootstock induced biotic response-related genes such as *NBS* and *NBS-LRR* type transcription factors and triggered a remarkable increase of stilbene synthase transcripts associated with ROS scavenging activity and oxidative stress prevention (Chitarra et al., 2017). Thus, specific rootstocks may induce biotic stress responses and confer resistance to disease through genome-wide modulation of the scion transcriptome.

Although most investigations have focused on the effect of rootstock on scion transcriptomes, studies on the effect of scion inducing gene expression changes in the rootstock remain limited. A very recent study by Gautier et al. (2020) investigated the control exerted by scions of grafted grapevine on rootstock transcriptomes and in particular under conditions of low phosphate. By employing an RNA-seq approach these authors examined rootstock transcriptome responses in different scion-rootstock genotype combinations of grapevine grafts in relation to low phosphate treatment and demonstrated differential transcriptome responses upon low phosphate imposition that depended on the particular rootstock-scion combinations (Gautier et al., 2020).

Notably, aside from transcripts, protein abundance differences depending on rootstock have also been reported. Increased salinity in grapevine cv. Thompson Seedless grafted onto a salt-tolerant rootstock (110R) is associated with differential protein accumulation involving photosynthesis, chlorophyll biosynthesis, amino acid metabolism, and precursor metabolite biosynthesis, as compared to the own-rooted plants (Patil et al., 2020).

Finally, molecular factors determining the success of graft healing and union formation in relation to graft compatibility/incompatibility have been investigated in grapevine. During the process of union formation in Cabernet-Sauvignon heterografts, CS/RG, and CS/1103 Paulsen, transcriptional changes were detected at the graft interphases of both combinations and these were associated to cell wall modification, wound responses, hormonal signaling, and stress responses, all playing a role in the complex mechanism of graft healing and development (Cookson et al., 2014). A very recent study utilized a transcriptomic approach with compatible and incompatible grapevine grafts of Touriga Nacional scions onto Richter 110 rootstocks to identify master regulators governing graft union formation. Grafts were analyzed in a nursery context at 21 and 80 days post-grafting (Assunção et al., 2019). Genes associated with hormone and metabolic signaling were markedly induced in the tissues of the graft zone in the compatible combination at the early stage whereas genes engaging in oxidative stress and wound healing showed higher induction in the less compatible combination at the later stage implying an inability of the later to cope with excess stress. In addition, reduced expression of specific miRNAs and concomitant increased expression of their target genes encoding transcription factors, which regulate cambium maintenance and vascular tissue differentiation was evident in the compatible grafts at the later stage (Assunção et al., 2019). Thus, the regulation of specific miRNA-target gene modules appears to be of high importance for proper graft union formation and could be of potential use for molecular prediction of graft success.

### Rosaceae

Grafting in fruit trees serves various purposes such as yield increase, control of growth, early transition to reproductive stage, improved economic quality, and tolerance to biotic and abiotic stresses. Grafting is widely used within the *Rosaceae* family members such as apple, pear, peach, and cherry which greatly contribute to a balanced and healthy diet (Farinati et al., 2017).

The underlying molecular mechanisms by which the rootstock controls scion growth and properties in *Rosaceae* remain largely unknown. Research has focused mainly on anatomical features, hormonal interactions, and nutrient transport across graft union (Sorce et al., 2002; Zamorskyi, 2011; Valverdi et al., 2019). Some preliminary studies aimed to identify miRNAs from vascular tissue and phloem sap that are possibly involved in long-distance signaling and modulation of expression and movement of mRNA targets (Varkonyi-Gasic et al., 2010). In grafted apple, the first reports on long distance RNA movement came in 2010 mainly focusing on their implications to hormone signaling (Kanehira et al., 2010). These authors revealed the transport of *MpSLR/IAA14* transcripts from the wild rootstock (*Malus* var. ringo Asami Mo84-A) to the scion of apple cv. Fuji by *in situ* hybridization in the phloem. The same year the transport of gibberellic acid insensitive mRNA transcripts (*GAI*) was detected in both directions (upward and downward) from the graft union (Xu et al., 2010). In a similar study, the transport of *Gibberellic acid insensitive* transcripts was demonstrated across the graft union from a wild *Pyrus betulaefolia* cv. Bunge (rootstock) to a traditional local Chinese pear cultivar, *Pyrus bretschneideri* cv. Yali (scion) in a distance up to 50 cm (2 years old grafting tree) (Zhang et al., 2012). Moreover, the transmission capacity of Pyrus *GAI* transcript was successfully tested by grafting a 35S:pear -*GAI* transgenic tobacco (*Nicotiana tabacum* L. cv. Samson.) to wild-type tobacco and detecting transmission of *GAI* mRNA through the graft union occurred soon as 15 days after grafting. Findings in arabidopsis demonstrate that trafficking of *GAI* RNA transcripts is mediated by specific RNA motifs (Huang and Yu, 2009); it remains to be verified whether these motifs are responsible for long distance signaling in fruit trees as well.

Few works have pointed out the involvement of long-distance signal molecules to biotic stress tolerance. Investigation of resistance mechanisms of three apple rootstocks to wooly apple aphid, showed that the resistance factors exist in the phloem tissue, implying the modulation of resistance via a long-distance signal (Sandanayaka et al., 2003). Kaja et al. (2015) studied the expression levels of miRNAs on four different apple rootstocks (two resistant and two susceptible in fire blight) by deep sequencing of 12 small RNA libraries. Their results together with stem loop qPCR analysis revealed four apple miRNAs that could be potentially involved in fire blight resistance (mdm-miR169a, mdm-miR160e, mdm-miR167b-g, and mdm-miR168a,b) (Kaja et al., 2015). Thus, they assumed that the miRNA expression profiles in scion could influence rootstock characteristics, such as resistance to this bacterial pathogen.

Recent experiments have aimed to unravel the role of transmissible signals and epigenetic mechanisms on flower induction. In a recent study, it was demonstrated that a *PbWoxT1* from pear (*Pyrus betulaefolia*) interacts with a polypyrimidine tract binding protein *PbPTB3* assisting in long distance transport in the phloem and possibly controlling flower development and growth (Duan et al., 2016). Fan et al. (2018) investigated the role of 12 histone modification (*HMs*) genes in flower induction in two apple varieties with contrasting flowering characteristics. Their expression patterns indicated that their up- or down-regulation contributed to different aspects of flowering. Although the two varieties were both grafted to the same rootstock, the relationship of grafting to the differential expression of the *HMs* was not investigated. An et al. (2018) studied the miRNA expression in self-rooted and grafted Fuji apple trees by high-throughput sequencing in relation to flowering rate. Some of them that were differentially regulated in response to grafting (miR156, miR172, miR159, miR171) target genes encoding transcription factors that regulate flower formation, and suggested that there is a regulatory network between miRNAs and mRNAs that is generated by grafting. Mir156 and mir172 are suggested to act as mobile signals regulating flower formation in grafted arabidopsis plants as well (Zhu and Helliwell, 2011; Wang, 2014).

Inversely, the role of scions onto rootstocks has been examined in a study by Li et al. (2016). Combined morphological, metabolic, hormonal, and root transcriptome analysis of *M. robusta* rootstock grafted with scions of wild-type (WT) apple (*M. spectabilis*) and a more-branching (MB) mutant at the branching stage revealed that sugar metabolism and auxin and cytokinin signaling exert major effects on root growth and development of grafted apple (Li et al., 2016).

Future work should enable to extend our limited so far knowledge about the implication of mobile signals in controlling agronomically important traits in grafted trees as well as to find commonalities and differences regarding their role in herbaceaus and woody grafted plants (Thomas and Frank, 2019).

### Other Woody Crop Plants

Studies with citrus grafts have shown differential DNA methylation in different scion/rootstock combinations. Valencia orange (VO) scion variety grafted on two rootstocks with different soil water extraction capacities, Rangpur Lime (RL) and Sunki Maravilha (SM), displayed differential graft-induced DNA methylation which were associated with enhanced acclimatization responses upon recurrent drought stress for the VO/SM combination (Neves et al., 2017). Similarly, “Tahiti acid lime” (TAL) scions grafted on RL and SM exhibited hypermethylation and hypomethylation, respectively, upon water deficit treatments, however, physiological responses were similar (Santos et al., 2020).

Ahsan et al. (2019) provided the first evidence of avocado inter-graft regulation of miR156 and miR172 that control transition from juvenile to flowering phase by mediating the expression of the *SQUAMOSA* promoter binding protein-like (SPL) gene using different combinations (scions from shoot of young seedlings or mature scion wood grafted onto both seedlings or clonal rootstocks). Results demonstrated that scion age significantly influenced grafted tree maturity through the control of miR156-*SPL4*-miR172 regulatory network. They also showed that the existence of leaves on cutting rootstocks provided grafting success and influence the abundance of miR156 and miR172 in the scion (Ahsan et al., 2019).

An interesting study conducted by Khaldun et al. (2016) examined the miRNA regulation and expression patterns of tomato plants grafted onto Goji Berry (*Lycium chinense* Mill.) to reveal their implication to diverse biological pathways within a distant-grafting system by using Illumina sequencing technology. Significant differences in miRNA expression of shoots and fruits were revealed comparing non-grafted to grafted tomato plants. The setting of fruits was found to be the stage with the highest abundance of miRNA transcripts (123 were up-regulated). Potential targets of differentially expressed miRNAs identified by *in silico* analysis were found to be mainly transcription factors involved in diverse metabolic and regulatory pathways. Global transcriptome changes have been detected also between compatible and incompatible graft combinations in *Litchi chinensis* (Chen et al., 2017) and during graft union development in pecan (*Carya illinoinensis*) (Mo et al., 2018).

### Forest Trees

Grafting has been used empirically also in clonal forestry section as a tool which primary targets transfer of juvenility properties to mature materials by establishing them onto young rootstocks in nurseries (Huang et al., 1992). Phenotypic traits (such as wood density, tree height, biomass production) in superior forest trees are linked to economic benefits but limitations such as time needed for maturity to be reached and ineffective vegetative propagation limits the production of this material. Furthermore, recent findings showed that epigenetic modifications which can act through grafting procedures can be responsible for phase change from juvenility to maturity (von Aderkas and Bonga, 2000).

In a recent work with Spanish red cedar (*Cedrela odorata* L.) authors showed the significant effect of elite genotype grafting in juvenility induction in mature material (Robert et al., 2020). Interestingly, this phase change requires a particular balance and distribution of plant growth regulators among specific cell layers in meristematic tissues, where a “nurse tissue” condition is observed upon grafting of juvenile cells on scion tissues (Wendling et al., 2014). Those effects are hard to detect due to the fact that probably they involve the participation of epigenetic shifts mediated by siRNAs (Molnar et al., 2010) and require further research.

In another study conducted for *Hevea brasilensis* methylation differences were detected between buds from a single own rooted polyembryony-derived seedling grafted to genetically divergent rootstock (Uthup et al., 2018). MSAP and bisulfite sequencing techniques revealed significant alterations in DNA methylation profiles between heterografts and the levels of those epigenetic rearrangements was found to depend on the degree of incompatibility between the rootstock and the scion.

## Transgrafting

**FIGURE 3:**
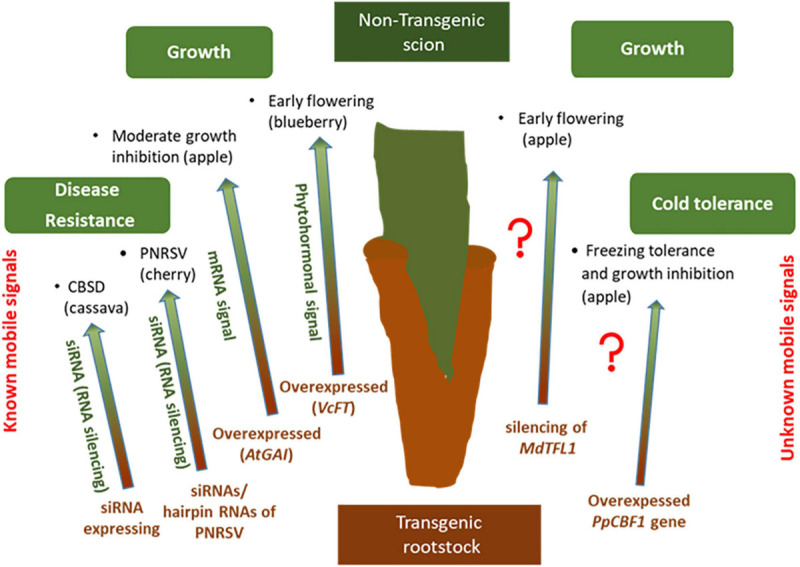
Examples of plant traits that were altered by transgrafting on woody plants (transgenic rootstock/non-transgenic scion) by known **(left)** and unknown **(right)** transmissible signals.

In grafted trees, the long-distance transport of signaling molecules through phloem and xylem, has proven as a major communication mechanism that fine-tunes tree architecture, developmental transitions, abiotic and biotic stress tolerance and cold-hardiness (Thomas and Frank, 2019; Figure 3). T he term trans-grafting is used when only one part (more often rootstock rather than scion) is transgenic with the other part untransformed (Haroldsen et al., 2012; Limera et al., 2017). The interaction of transgenic and non-transgenic parts in trans-grafted plants through the translocation of mRNA and RNAi (small RNA interference) molecules via the vascular system is the core of trans-grafting technique (Haroldsen et al., 2012). Consequently, and particularly in fruit crops, trans-grafting provides the potential improvement of woody tree species without the need of long-lasting biosafety controls normally required for traditional genetically modified plants (Song et al., 2019a).

To date, several studies focused on movement of RNAi- based silencing molecules from transgenic rootstock to non-transgenic scions to enhance pathogen resistance in perennial woody plants (Lemgo et al., 2013). The first study investigating the impact of long-distance-moving signals on conferring disease resistance was conducted by Dutt et al. (2007), where they identified that Shiva-1, a small lytic peptide, is transmitted from transgenic rootstocks to scions in grapevine and subsequently control the spreading of the xylem limited bacteria *Xylella fastidiosa*, which also protects from Pierce’s disease (PD). Transgenic cassava rootstocks expressing sRNAs in order to trigger RNA silencing in non-transgenic scions have successfully prevented the spreading of pomovirus Cassava brown streak disease (CBSD) (Yadav et al., 2011). Moreover, transgenic cherry rootstocks containing a hairpin RNAi vector were capable of combating *Prunus* necrotic ringspot virus (PNRSV) (Song et al., 2013). Later on, it was demonstrated that long-distance transportation from transgenic rootstock to non-transgenic sweet cherry scions of small interfering RNAs (siRNAs) derived from short hairpin RNAs of PNRSV induced systemic silencing and improved protection against PNRSV (Zhao and Song, 2014).

However not all of RNAi-based rootstocks can efficiently transfer the silencing molecules to non-transformed scions and confer resistance. In another study, hairpin siRNA derived from transgenic rootstocks of lime plants did not confer resistance to tristeza tree virus (CTV) in the non-transgenic scions, mainly due to the fact that the biggest fraction of siRNA was very quickly degraded (Lopez et al., 2010).

Grafting of transgenic rootstocks produced mRNA and siRNA signals and was also tested for speeding-up transition to flowering stage and for controlling tree size, characteristics that are useful for agriculture (McGarry and Kragler, 2013; Song et al., 2015). Interestingly, none of these studies confirmed the transmission of a long-distance mRNA or siRNA signal. In the work described by Flachowsky et al. (2012) attempted to downregulate the *Terminal Flower1* (*MdTFL1)* gene, in order to induce early transition from juvenile to flowering stage in apple trees. However, when dsRNAi*MdTFL1* transgenic rootstocks were grafted onto non-transgenic apple genotype “PinS,” the flower-inducing signal obtained after silencing of *MdTFL1* gene was not transmittable (Flachowsky et al., 2012). A study by Smolka et al. (2010) aimed to reduce vegetative growth of apple trees by using transgenic rootstocks carrying the root-inducing *rolB* gene of *Agrobacterium rhizogenes*. Although these transgenic apple rootstocks significantly altered the characteristics of the scion when cultivated in nursery, no transmission of big molecules such as *rolB* gene or its mRNA was detectable in the scion cultivars. Moreover, overexpression of a peach *CBF* (*PpCBF1*) gene in a transgenic apple rootstock, responsible for enhanced freezing tolerance and growth inhibition, provoked reduced scion growth and delayed flowering with no evidence of phloem-transmissible *PpCBF1* mRNA (Artlip et al., 2016). A transgenic apple rootstock variety, *Malus prunifolia*, integrating the *AtGAI* gene reduced moderately the growth rate of the Malus cultivar “Orin” (Xu et al., 2013).

A very interesting study by Song et al. (2019b) showed that transgenic blueberry rootstocks overexpressing flowering locus T gene (*VcFT)* induced early flowering in non-transgenic scions and the mobile signal responsible for this was a transmissible phytohormonal signal. Finally, in some woody tree species such as nut crops transgrafting has been applied very recently but with encouraging results, confirming its emerging role in tree breeding (Liu et al., 2017).

## Implications for Crop Improvement

It is anticipated that in coming years plants will face major environmental challenges such as rapid rise in temperature, increased drought conditions and extreme weather phenomena. The impact of biotic stresses will be exacerbated owing to regional climate changes favoring a variety of pathogens. These factors are expected to have considerable impacts in crop productivity and food security. Therefore, the development of new agricultural practices or the capitalization of existing technologies is an imperative for confronting the above threats and imparting crops with resilience to unfavorable climatic conditions.

Grafting is an ancient agricultural practice originally used to counteract soil borne diseases, and ameliorate the performance of woody fruit species, enhancing yield, fruit quality, and tolerance to external stressors. Despite its widespread use worldwide the molecular mechanisms governing grafting and scion-rootstock communication are still not well understood. Progress has been achieved by research in model and vegetable plants that has contributed to a better insight into graft regulation at the molecular level. Lately this research has been extended to woody species. It is more and more evident that graft success and proper development of the grafted plant resides on scion-rootstock communication that involves epigenetic interactions and transcriptional modulation. mRNAs, miRNAs, and proteins can act as potential short- and long-distance signals exerting a regulatory role in the receiving graft partner which impacts scion but also whole plant performance. In grapevine, apple and avocado, miRNA and mRNA molecules as well as induction of transcriptional reprogramming in scions has been associated with physiological and metabolic pathways affecting traits of agronomical relevance (Cookson et al., 2014; Kaja et al., 2015; Corso et al., 2016; Li et al., 2016; Chitarra et al., 2017; Pagliarani et al., 2017; An et al., 2018). Gaining a deeper insight into the molecular mechanisms underlying these processes could direct the development of suitable molecular markers to be used for generating and selecting superior grafts with improved yield, quality, and tolerance to abiotic and biotic stresses.

Furthermore, graft compatibility is a prerequisite for graft success. Studies have started to explore the molecular regulators determining rootstock-scion compatibility (Cookson et al., 2014; Mo et al., 2018; Assunção et al., 2019; Notaguchi et al., 2020).

This information can be utilized to develop molecular markers for detecting successful grafts at early stages of graft development. Such diagnostic biomarker tools would be of great value to nurseries for early prediction of successful grafting and rapid and efficient selection of superior graft combinations, something particularly important for woody species where incompatibility may not be apparent before a period of several years.

Moreover, trans-grafting technology has enabled the grafting of wild type scions onto genetically engineered rootstocks carrying desirable traits, circumventing genetic modification of the scion. Thus, the transgene is contained in the rootstock and provides its beneficial effects such as resistance to viruses and other detrimental disease as well as tolerance to abiotic stressors, without genetic modification of the product or edible fruit (Zhao and Song, 2014; Artlip et al., 2016; Song et al., 2019b). This promising technology holds great potential as a targeted approach in woody plant improvement.

Finally, epigenetic factors may play an important role in improvement of woody plants through grafting. Epigenetic changes such as DNA methylation, histone modifications and the action of small RNAs can result in gene expression changes without affecting the underlying DNA sequence, with major impacts in cellular function and subsequent plant development and performance. In certain occasions these changes may be transmitted to the next generation. Stably inherited natural, environmental or graft-induced epigenetic changes have the potential to generate epi-alleles and lead to phenotypic variation (Gallusci et al., 2017; Kapazoglou et al., 2018; Varotto et al., 2020). In the context of changing environments, transgenerational effects, transgenerational memory, or transgenerational inheritance is the phenomenon that is defined as the “memory” at molecular level of the environmental conditions that an organism experienced which leads to modification of progeny phenotype (Tricker, 2015; Bilichak and Kovalchuk, 2016). This means that plants remember the stress they have experienced during their lifetime and transfer this memory to their offspring. As a result, offspring presents with increased resilience to stress exposure as compared to their parents. To date, the molecular base of this phenomenon is not fully understood. It seems that during gametogenesis some epigenetic marks escape the process of epigenetic reprogramming and resetting and subsequently are passed on to the offspring (Bilichak and Kovalchuk, 2016). The research about the transgenerational effects in plants has been focused mostly on annual plant species (Suter and Widmer, 2013; Zheng et al., 2013; Herman and Sultan, 2016) whereas studies in herbaceous perennial species reported that they might adopt a sexual reproductive strategy (Latzel et al., 2014) or an asexual reproductive strategy (Ren et al., 2017; Rendina González et al., 2018). Inversely, very limited information is available about the woody long-lived perennial species and especially those whose commercial varieties are maintained by vegetative (clonal) propagation. Long-lived perennial species potentially can accumulate epigenetic modifications during their lifetime that may be transferred to their offspring. Unlike sexually reproduced species where epigenetic modifications could be lost during meiosis as a result of erasure and resetting of epigenetic marks the possibility of stable transmission of epigenetic changes may be increased in asexually propagated plants as only mitotic cell divisions are involved (Verhoeven and Preite, 2014; He and Li, 2018).

In this regard, a recent report described putative transgenerational effects at the DNA methylation level as a result of temperature and photoperiod experienced by mother plants on the bud phenology of asexually produced offspring of *Populus deltoides* and *Populus trichocarpa* (Dewan et al., 2018). These authors underlined the importance of this effect on the potential response of plants to climatic changes. In another study, Perrin et al. (2020) compared the DNA methylation pattern in apples (*Malus domestica*) between adult trees and seedlings from selfing as well as between mature donor trees and newly grafted trees (Perrin et al., 2020). According to their results, in the former comparison the DNA methylation pattern did not differ at whole genome level but clearly varied in regions with genes related to photosynthesis. On the other hand, in the latter comparison, the majority of DNA methylation patterns was transferred from donor trees to newly grafted trees. They concluded that from a physiological, transcriptomic, and epigenomic point of view, newly grafted plants are in a phase between an adult tree and a seedling (Perrin et al., 2020). The above findings together with the fact that epigenetic variation contributes significantly to phenotypic plasticity, especially in species with limited genetic variation, points to the benefits of exploiting the potential of epigenetic variability. Further understanding of the molecular mechanisms regulating epigenetic changes such as DNA methylation in woody plants will contribute to the development of diagnostic molecular epi-markers for selection of superior genotypes and grafts with advantageous traits of agronomic relevance.

## Conclusion and Future Prospects

The ongoing climate change and rapid expansion of global population growth call for concerted efforts by diverse fields in order to mitigate the effects of adverse climatic conditions, promote crop sustainability, ensure food security and enable preservation of biodiversity.

In this context, woody species grafting is a promising agriculture technology for generating improved woody plants that can face environmental challenges without major compromise in yield and quality and with low input requirements. Despite its widespread use and the wealth of knowledge concerning woody species grafting at the morpho-physiological level, little is known about the molecular mechanisms governing this process. Nevertheless, research has now begun to address the molecular aspects associated with woody species scion-rootstock compatibility and successful graft development.

For some species like grapevine and apple, progress has been accomplished in terms of the graft-triggered transport of small RNAs or mRNAs long-distance between the graft partners and the characteristics they impart on the scion or whole grafted plant. In addition, RNA seq technologies have revealed graft-induced transcriptome reprogramming in scions with potential impact on phenotype. Epigenetic changes such as altered DNA methylation or histone modifications in woody species, is an area largely unexplored but in some cases like apple, citrus, avocado, and rubber tree investigations have started on DNA methylation alterations associated to grafting. There are no reports thus far on graft-induced histone modifications which is another crucial epigenetic mark that regulates cell function and plant development. Investigations on DNA methylation and histone modifications should be the focus of future studies in order to delineate further the molecular mechanism of grafting.

Moreover, trans-grafting technology has allowed targeted manipulation of rootstock genes affecting important scion traits, but without transferring the modified gene to the scion. This technology is gaining ground and holds a lot of potential toward generating grafts of agronomically important woody crops with improved qualities.

Challenges ahead include deeper understanding of the genetic and epigenetic molecular mechanisms underlying graft compatibility and proper growth and resilience of the grafted woody plants. This will contribute significantly to our knowledge on the molecular factors regulating these processes. Consequently, it will direct the development of molecular markers and epi-markers for rapid and efficient detection of graft success and compatible graft combinations at early stages as well as selection of superior grafts with improved adaptability to environmental challenges.

Finally, adaptive transgenerational plasticity and the molecular mechanisms governing this phenomenon is an under-explored area with great potential for agricultural application in woody plants. Graft induced-epigenetic variants (epi-allelles) are more likely to be stably inherited across generations in woody perennials than vegetables due to their way of clonal propagation (asexual reproduction) which favors the stable transmission of new characters. It is anticipated that in coming years research will expand greatly in uncovering mechanisms associated with woody plant grafting and transgenerational heritable changes. This knowledge would be of utmost importance for generating and selecting superior grafts for woody crop improvement and for transmitting graft-induced beneficial traits to subsequent generations, in order to meet sustainability demands in a changing environment.

## Author Contributions

AK coordinated author contributions, contributed with specific parts, and edited the manuscript. ET, EVA, EMA, MG, SM, and GD contributed with specific parts each. AD had the idea and edited the manuscript. All authors contributed to the article and approved the submitted version.

## Conflict of Interest

The authors declare that the research was conducted in the absence of any commercial or financial relationships that could be construed as a potential conflict of interest.
